# Topical Ketamine with Other Adjuvants: Underutilized for Refractory Cancer Pain? A Case Series and Suggested Revision of the World Health Organization Stepladder for Cancer Pain

**DOI:** 10.1089/jpm.2019.0618

**Published:** 2020-08-19

**Authors:** Jennifer A. Winegarden, Daniel B. Carr, Ylisabyth S. Bradshaw

**Affiliations:** ^1^ Department of Medicine, The Medical Team Hospice, Livonia, Michigan, USA.; ^2^ Department of Public Health and Community Medicine, Pain Research, Education and Policy Program, Tufts University School of Medicine, Boston, Massachusetts, USA.

**Keywords:** adjuvants, cancer, ketamine, pain, topical

## Abstract

***Background:*** Uncontrolled cancer pain is a significant problem in palliative medicine. Opioids are often first-line treatment that increase risks of analgesic tolerance and hyperalgesia. Topical ketamine with other adjuvant pain medications is an often-overlooked treatment, yet may be most effective in difficult-to-treat cancer pain.

***Objective:*** We report a case series of hospice patients with uncontrolled cancer pain who were suboptimally treated with opioids and nerve blocks, whose symptoms responded to topical ketamine with other adjuvants. We review the pronociceptive properties of opioids and how topical multimodal treatment of cancer pain can be more effective than standard opioids, other topical adjuvant medications, and nerve blocks. We discuss the shortcomings of the World Health Organization (WHO) stepladder for the treatment of cancer pain and suggest an adjuvant treatment algorithm, directing physicians to appropriate adjuvant pain agents based on pain type and distinct receptor actions.

***Design:*** This is a retrospective case series of patients who responded to topical multimodal pain treatment with implementation of findings into an addendum to the WHO stepladder.

***Subjects:*** Subjects were from a case series of community-based hospice patients with previously uncontrolled cancer pain.

***Measurement:*** Measurement was made by self-report of pain levels using the 10-point numeric pain rating scale.

***Results:*** Patients' pain was controlled with topical adjuvant medications with return to previously lost function and prevention of otherwise escalating opioid dosing.

***Conclusions:*** These patient cases reveal how ketamine-based topical treatment for cancer pain can be more effective than standard opioids, other topical adjuvant medications, and nerve blocks with no noted side effects and observed reduction in opioid consumption.

## Introduction: Problems with Opioids and the WHO Cancer Pain Treatment Stepladder

Among other safety and efficacy concerns^1^ with the use of opioids in hospice and palliative care are the linked phenomena of analgesic tolerance and hyperalgesia. Other concerns involve the accumulation of bioactive metabolites of opioids, particularly when high opioid doses are given chronically. For example, morphine-3-glucuronide is pronociceptive and can lead to liver and kidney toxicity.^2,3^ Hyperalgesia during ongoing opioid usage reflects opioids' activation of the *N*-methyl-d-aspartate receptor (NMDAR), as well as stimulation of astrocytes.^4,5^ Because opioids play a central role in the World Health Organization's (WHO's) stepwise approach to cancer pain control, clinicians and researchers^6^ have been eager to explore nonopioid adjuvants or alternatives to opioids. A byproduct of shifting to opioid alternatives is the reduction of unmonitored at-home storage and administration of opioids, in which context they may be diverted and contribute to the current epidemic of opioid use disorder.

In light of the undesired effects of chronically administered opioids, and the need to balance responses to opioid tolerance and uncontrolled pain by increasing opioid doses with the risks of opioid hyperalgesia, clinicians struggle to provide analgesia for severe cancer pain in the face of otherwise shrinking options. We describe three hospice patients in whom adherence to the WHO analgesic ladder proved inadequate for pain control despite escalating doses of strong opioids, who showed prompt and dramatic analgesic responses after the addition of topical ketamine coformulated with other adjuvant pain medications.

## The WHO Analgesic Ladder: Critique

In 1986, the WHO developed a three-step “analgesic ladder” to meet the therapeutic challenges presented by severe cancer pain.^7^ Recognition of the already described mechanisms of opioid adverse effects and their clinical correlation occurred after the development and promulgation of this ladder.

Another weakness of this stepladder is a failure to differentiate different mechanisms of pain experienced in cancer and to describe adjuvant medications and routes most appropriate for each type. Thus, physicians have long depended on their familiarity with opioids, underutilizing other treatment options such as adjuvant pain medications. Descriptors of the quality, potential source, and mechanism of the pain, not just its severity, guide the choice of adjuvant medications.^8,9^

## Ketamine: A Valuable Adjuvant

Of the available adjuvant medications, ketamine is a powerful NMDAR antagonist. It also reduces production of inflammatory mediators; blocks uptake of dopamine, serotonin, and noradrenaline; decreases activation of microglia; blocks ion channels; and binds opioid receptors.^10^ Although developed as an anesthetic, ketamine at subanesthetic doses can reverse pain crises in patients taking opioid medications^11^ and also reverses opioid-induced neurotoxicity.^12^ Through multiple routes, with and without other adjuvant pain medications, ketamine's varied actions have been exploited to optimize cancer pain control.

## Incorporating Ketamine and Other Adjuvants into the WHO Ladder

Integrating adjuvant pain medications in routine practice may be facilitated by amending a familiar clinical decision aid such as the WHO ladder. Although other authors have suggested modification of this ladder,^13^ their suggestions have emphasized inclusion of costly high-tech interventions such as intrathecal pumps, rhizotomy, and neurolytic blocks.^14^ Advantages in using ketamine include its ability to inhibit spinal windup and central sensitization, providing a stabilizing and even preventative intervention; in addition, its cost is modest, particularly compared with more interventional procedures. The coadministration with other adjuvants, such as clonidine and gabapentin, can provide a more robust response, and formulation in Lipoderm base increases transdermal penetration.^15^

## Case Series

### Case 1

CK, a 98-year-old Caucasian female, was diagnosed with squamous cell vulvar cancer in July 2016. Her treatment was regional radiotherapy. After treatment, her physicians reported that the cancer was cured, and the patient experienced several months of only minimal discomfort attributed to radiotherapy. However, six months later, the patient began experiencing increased pain in the vulvar region. Although the patient was convinced that the cancer had returned, her treating physicians told her that was highly unlikely. As a result, the patient did not receive further evaluation until the recurrent cancer had extended dorsally to the rectum and superiorly to the bowel, when she was referred for palliative care.

The patient's pain had steadily increased, particularly in the superficial areas of the vulva, perineum, and rectal regions. Upon examination, the area was excoriated and had malignant-appearing lesions. As the patient was not a candidate for further aggressive therapy, her treatment focused upon pain relief. Tramadol provided minimal relief and intolerable dyspepsia. Hydrocodone-acetaminophen was poorly effective and caused hallucinations. The patient saw a pain specialist who performed a neurolytic ganglion impar nerve block with 10% phenol. This provided good short-term relief of pain but by three months later her pain returned. A subsequent right pudendal nerve block with 0.25% bupivacaine and 40 mg of methylprednisolone provided no relief. Topical benzocaine gel was ineffective. Lidocaine 5% gel applied to her perineum every one to two hours reduced her pain intensity slightly, but it was still severe (“9/10”). The pain was so severe that the patient attempted suicide by overdose of tramadol with an uncomplicated recovery in the hospital. Sitting upright or standing were hindered by pain. Toileting resulted in a searing pain persisting as long as hours.

The patient experienced progressive weakness, poor appetite, weight loss, vulvodynia, and pudendal neuralgia. In May 2018, the patient was admitted to hospice services. The lidocaine 5% gel was discontinued, and the patient was started on a topical Lipoderm cream containing ketamine 10%, clonidine 0.2 mg/mL, and gabapentin 6 mg/mL. The patient applied 3 mL of the cream to the affected area every 8 hours. She obtained near-complete pain relief within the first 30 minutes of applying the compound; however, the pain relief only lasted for 1–2 hours. Morphine sulfate immediate release (MSIR) was initiated at a dose of 7.5 mg PO (by mouth) every 4 hours as needed. This provided no analgesic benefit and made the patient feel “groggy.” The compounded cream was then increased to a frequency of 3 mL every 4 hours. Again, the patient experienced pain relief but had symptom breakthrough at two hours after application. Oxycodone 5 mg PO q 4 hours was added, in the place of the MSIR, but the patient experienced the side effect of disorientation without providing significant pain relief.

It was clear that the patient could achieve pain control through topical adjuvant pain medications, but that longer acting agents were needed. Based upon the availability of a longer acting local anesthetic (bupivacaine) and opioid (methadone), a modified Lipoderm cream was compounded with ketamine 10%, clonidine 0.2 mg/mL, gabapentin 6 mg/mL, bupivacaine 0.2 mg/mL, and methadone 0.2 mg/mL; 3 mL to be applied to the perineum every 4 hours. Within 30 minutes after the first application, this augmented medication provided nearly complete relief with a pain intensity <3 out of 10. She was able to resume normal posturing, toileting, and other activities of daily living without any pain. Comfort was maintained in the perineum and all adjoining involved areas, throughout the remainder of the patient's hospice course of 17 days. The patient's and family goals of care were achieved with use of the topical cream including comfort whether seated, lying, or standing, and with toileting.

### Case 2

JG, a 69-year-old Caucasian male, was admitted to hospice services with adenocarcinoma of the prostate diagnosed 2 years earlier, as stage IV (Gleason score 9), with diffuse metastases to bones including axial skeleton and the skull. The patient had received 6 cycles of docetaxel and 10 fractions of radiation therapy to the left posterior ribs. After completing these initial treatments, he continued with oral hormone-based chemotherapy, bicalutamide, and an injectable monoclonal antibody, denosumab, for bone lesion control and comfort. Despite these therapies, the patient's cancer continued to progress.

Over the six months before hospice admission, the patient had increasing pain, weakness, and fatigue. He had a 6-month weight loss of 21 kg, from initial weight of 77 kg, and was profoundly weak. He had daily symptoms of pain, nausea, flushing, depression, and insomnia. On admission to hospice, the patient was spending much of the day in bed with increasing hip and back pain, and fatigue. He was taking morphine sulfate extended release 30 mg orally every 12 hours, and oxycodone 5 mg orally every 6 hours as needed for pain. His pain was self-reported as between “8–10/10.” It was not uncommon for the patient to state his pain was “excruciating.” He described his pain as “deep,” “aching,” and “shooting.” He stated it was in the bilateral hips, low back, and pelvis. The patient did not have specific treatment for bone pain, despite diffuse bony metastases; therefore, oral dexamethasone was started immediately at 2 mg daily, given with his first meal, upon hospice admission.^18,19^ The patient noted significantly improved comfort and enjoyed increased activity and appetite.

The patient's pain then varied over the next seven months on hospice requiring multiple titrations to his oral pain regimen. His dexamethasone had been increased incrementally to a final oral dosing of 8 mg daily, given with his first meal, with continued control of bony pain. His baseline opioid was changed from morphine sulfate extended release 30 mg BID (twice daily) to methadone (for continued mu-agonism and partial NMDAR antagonism)^17^ with a final dose of 30 mg QID (4 times daily) in the final 2 weeks of his life. The patient's oxycodone/acetaminophen was increased from 5/325 to 10/325 and was given 1–2 PO q 4 hours in the final 2 weeks of his life. In addition, the patient admitted that he had existential pain but continued to refuse spiritual care counseling throughout his hospice course.

During this seven-month period, the patient was developing symptoms of cognitive impairment and severe bony pain of the left mandibular and orbital regions. The major contributors to his pain and suffering were multiple masses originating from below the left mandible and left cheek. He could no longer tolerate dentures and was placed on a liquid diet due to odynophagia. Then a topical Lipoderm cream of ketamine 10%, clonidine 0.2 mg/mL, and gabapentin 4 mg/mL was initiated with a schedule of 1.0 mL to the affected area every 8 hours. The patient had rated his pain intensity as “8/10” at the time of the first application of cream. At 30 minutes after administration, the patient described “some improvement.” By 60 minutes after the first dose, the patient rated his pain intensity at “4/10” with this intervention alone. Between the 8-hourly applications of cream, his mandibular pain would reach a maximum of “4–5/10” but application of the next dose would reduce the pain to “2–3/10.” The patient resumed eating and denied mandibular pain.

Two weeks after the painful masses noted in the left mandible and cheek, additional masses grew at the inferior and right side of the mandible. The cream was then applied at 1.0 mL bilaterally TID with good relief of pain symptoms.

In the last 2 weeks of his life, the frequency of cream application was increased to 1.0 mL every 4 hours bilaterally. This controlled the mandibular and facial pain throughout his end of life, allowing the patient to eat and achieve goals of care.

### Case 3

MZ, a 72-year-old Caucasian female, was admitted to hospice services with ovarian carcinosarcoma diagnosed 3 years prior as stage IIIC, with disseminated malignant metastases to the retroperitoneum, peritoneum, and colon. She underwent radical debulking surgery and chemotherapy with carboplatin and five cycles of Taxol followed by carboplatin and docetaxel for eight cycles. She achieved a short remission. Four months later, the ovarian cancer returned with left adnexal mass, and the patient had surgical removal with a colostomy placed and further debulking surgical procedures. The patient was then treated with 5 cycles of ifosfamide and paclitaxel with 14 days of palliative radiation therapy for severe “10/10” pain of the coccyx. After 2 years of continued cancer presence, the patient presented to hospice services with a recurrence of the ovarian carcinosarcoma, and an additional primary cancer of the right upper lung with likely metastasis to the left axilla, and new metastasis to the coccyx: all within the previous 12 months. The patient was admitted to hospice with symptoms of anorexia-cachexia syndrome, nausea, intermittent shortness of breath, and pain.

The patient's pain was mainly movement related. She was taking pregabalin 50 mg twice daily and using a fentanyl 50 mcg/hour patch but was suffering with upper back pain; a sharp radiating pain initiating in the right back and wrapping around to the anterior ribs, as well as lower extremity chemotherapy-induced polyneuropathy. The patient started methadone 5 mg BID and the fentanyl patch and pregabalin were discontinued 5 days later. The patient's baseline pain improved but she had intermittent breakthrough pain. The methadone was increased to TID after five days with good results initially. For improved coverage of neuropathic and nociceptive pain, oral ketamine was added to the methadone 2 weeks later at 10 mg BID. The ketamine was helpful, but again the patient had ongoing breakthrough pain episodes. The oral ketamine was increased to 10 mg TID but the patient experienced one distressing vivid dream. The oral ketamine was then reduced to 5 mg TID, which was effective for her localized (back) and generalized pain.

At the time of hospice admission, the left axillary nodule was <2 cm. It was asymptomatic initially; however, it was doubling in size every one to two weeks. As the lesion grew rapidly beyond 3 cm, the patient developed lesion-associated severe pruritis, which was understood to be a pain equivalent. A Lipoderm cream of ketamine 10%, clonidine 0.2 mg/mL, and gabapentin 4 mg/mL was prescribed for the axillary lesion initially: 1.0 mL TID. The pruritis-associated discomfort decreased from “severe” to “none,” or “mild.”

The patient also developed a severely painful lesion of the left labium for which a work-up was declined. The lesion grew rapidly and was accompanied by vaginal bleeding, with severe sharp and burning pain, and was assumed to be malignant. During a vaginal pain crisis, the ketamine, clonidine, and gabapentin cream being used in the treatment of the left axilla area was also applied to the perineal region. The pain of the labial lesion decreased from “10/10” to “6/10” within 20 minutes after the cream was applied. At that point, the treatment was increased to 1.0 mL TID applied to both the left axillary and left labial mass. The dosing remained at this level until 10 days before the patient's death when it was increased to six times daily to both areas with control of the left axillary pruritis and the left labial pain.

Finally, as the axillary mass grew to a size of ∼14 × 8 × 5 cm, the patient developed a nonpruritic pain that was not improved by the ketamine, clonidine, and gabapentin cream; therefore, the cream was discontinued to the axilla but remained effective for the labial pain.

As the patient neared death, her “all over” pain again increased to 5/10. In the final three days her pain medication was titrated again. Her final doses were methadone 10 mg PO QID, ketamine oral solution 5 mg PO TID, and hydromorphone 2 mg PO every 2 hours PRN (as needed), with excellent control of generalized pain. The ketamine, clonidine, and gabapentin cream remained at 1.0 mL every 4 hours to the left labia with pain controlled despite continued growth of the labial mass. The patient died peacefully at home, with relief of symptoms, as was her wish.

## Discussion: The Need to Recognize Topical Adjuvant Analgesics in the WHO Method

In light of the increasing obstacles to opioid availability and the undesired side effects of chronically administered opioids for any diagnosis, clinicians struggle to provide analgesia for severe cancer pain. We suggest an addendum to the WHO stepladder to choose adjuvant medications based on efficacy, mechanisms of action, and routes of administration. An expanded treatment algorithm may be beneficial as an aid to clinical decision making for practitioners to select appropriate adjuvant pain agents based on their distinct receptor actions. Choices include three of the most commonly used topical analgesics for chronic neuropathic pain: ketamine, clonidine, and gabapentin, with ketamine having the highest absorption rate.^15^

Clonidine has been used as an adjuvant pain medication successfully in postoperative and chronic pain. As a relatively nonspecific α_2_-agonist, it acts to hyperpolarize nerve cell membranes, diminishing nociceptive function, and lowering circulating levels of catecholamines.

Gabapentinoids, through their action on the α2δ-1 C terminus of the NMDAR, modulate synaptic transmission, reversing the synaptic NMDAR hyperactivity caused by neuropathic pain, and normalizing nerve injury-induced activity of the spinal dorsal horn.^16^

This small case series describes topical treatment for neuropathic, nociceptive, and inflammatory pain in patients with end-stage cancer. For these patients, topical treatment allowed for control of symptoms where standard opioids, other topical adjuvant medications, and nerve blocks had failed. These findings are consistent with results in our noncancer palliative care patients with similar pain issues.

## Conclusions: Topical Multimodal Pain Therapies Effective in Uncontrolled Cancer Pain

**FIG. 1.**
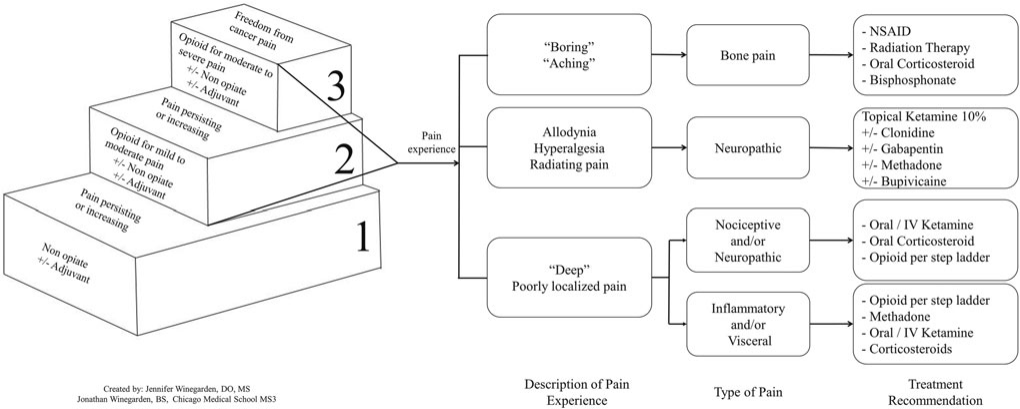
Addendum to the 1986 WHO cancer pain relief stepladder. WHO, World Health Organization.^7,10,13,15,17–19^

Given our consistent findings in palliative care cases with cancer-related pain, the following recommendation to the current WHO stepladder for cancer-related pain is suggested (Fig. 1). This algorithm provides suggestions for clinicians based on the type/descriptor of the pain, its location related to body surface, and correlation of the medication therapies to the patient's pain experience. These recommendations will help clinicians more accurately and confidently create patient-specific treatment plans that are inclusive of both traditional opioid treatment and multimodal adjuvant pain therapy. This alternative ladder may be best evaluated in a more formal way prospectively or tried when adherence to the WHO ladder fails to achieve a good effect/adverse effect ratio.

In light of the continued opioid crisis, randomized controlled trials are needed in the use of ketamine as a supplement or substitute for systemic opioids. These studies should explore topical routes where primary afferent neurons are easily accessible and acquire data to allow identification of patients more or less likely to benefit. There is a need for a systematic multicenter site trial to further assess the efficacy and safety of ketamine, with or without other adjuvant medications, for topical analgesia in uncontrolled cancer-related pain.
